# Why mothers still deliver at home: understanding factors associated with home deliveries and cultural practices in rural coastal Kenya, a cross-section study

**DOI:** 10.1186/s12889-016-2780-z

**Published:** 2016-02-03

**Authors:** Rodgers O. Moindi, Moses M. Ngari, Venny C. S. Nyambati, Charles Mbakaya

**Affiliations:** 1 Jomo Kenyatta University of Agriculture and Technology, Nairobi, Kenya; 2 Kenya Medical Research Institute/Wellcome Trust Research Programme, Centre for Geographic Medicine Research-coast (CGMRC), Kilifi, Kenya; 3 Kenya Medical Research Institute, Nairobi, Kenya

**Keywords:** Home delivery, Maternal mortality rate, Delivery practices, Cultural factors, Skilled delivery assistance

## Abstract

**Background:**

Maternal mortality has declined by 43 % globally between 1990 and 2013, a reduction that was insufficient to achieve the 75 % reduction target by millennium development goal (MDG) five. Kenya recorded a decline of 18 % from 490 deaths in 1990 to 400 deaths per 100,000 live births in 2013. Delivering at home, is associated with higher risk of maternal deaths, therefore reducing number of home deliveries is important to improve maternal health. In this study, we aimed at establishing the proportion of home deliveries and evaluating factors associated with home deliveries in Kilifi County.

**Methods:**

The study was conducted among mothers seeking immunization services in selected health facilities within Kilifi County using Semi-structured questionnaires administered through face to face oral interviews to collect both quantitative and qualitative data. Six Focus Group Discussion (FGD) and ten in-depth interviews (IDIs) were used to collect qualitative data. A random sample of 379 mothers was sufficient to answer the study question. Log-binomial regression model was used to identify factors associated with childbirth at home.

**Results:**

A total of 103 (26 %) mothers delivered at home. From the univariate analysis, both mother and the partners old age, being in a polygamy marriage, being a mother of at least two children and staying ≥5 Kms radius from the nearest health facility were associated with higher risk of delivering at home (crude *P <* 0.05). Both mother and partner’s higher education level were associated with a protective effect on the risk of delivering at home (RR < 1.0 and *P <* 0.05). In multivariate regression model, only long distance (≥10Kms) from the nearest health facility was associated with higher risk of delivering at home (adjusted RR 3.86, 95 % CI 2.13 to 7.02).

**Conclusion:**

From this population, the major reason why mothers still deliver at home is the long distance from nearest health facility. To reduce maternal mortality, access to health facility by pregnant mothers need to be improved.

## Background

Kenya has made progress towards reducing maternal mortality, though insufficient to achieve MDG 5 [1]. Nearly all maternal deaths can be prevented if mothers could deliver at a health facility under care of skilled birth attendant [2]. The presence of skilled birth attendant during childbirth in a hygienic environment with necessary skills and equipment to recognize and manage any emerging complications reduces the likelihood of birth complications, infections or death of either the baby or mother. According to the 2008–09 Kenya Demographic and Health Survey (KDHS) [3], more mothers died during childbirth in 2008–09 compared to 2003 [4]; 488 deaths in 2008–09 vs. 412 deaths per 100,000 live births in 2003. There was not much change on the proportion of women delivering in health facility under watch of a skilled birth attendant from 2003 (42 %) to 2008 (44 %), however in 2014, 62 % of deliveries were attended by skilled birth attendant in a health facility [5] In Kilifi County, only 52.3 % of all women who gave birth were attended by skilled birth attendant at a health facility in 2014 [5].

The Kenya government has rolled up many interventions and policies to ensure all childbirth is in a health facility and attended by skilled birth attendants. These policies and interventions include making child delivery in public health facility free since 2013, putting up of a maternal shelter (commonly referred to as ‘waiting homes’) that is currently underutilized, Output Based Approach (OBA) and the ‘Beyond zero campaign’ spearheaded by the first lady to stop preventable maternal deaths by providing fully equipped mobile clinic. OBA projects have been running in Kenya since 2006 targeting subsidies for safe motherhood in many parts of the country, with Kilifi County as one of them but their major weakness has been overreliance on external funding [6]. In addition, the County government and the national government via constituency development funds have built new health facilities. The uptake of antenatal care services (ANC) has been impressive in the Kilifi County, but seems like mothers opt to deliver at home after attending the ANC and later have their babies receive immunizations in the health facilities [5]. Despite these deliberate interventions by the government, it’s still not clear why mothers would opt to deliver at home. Therefore, this study seeks to understand what makes mothers deliver at home, home delivery cultural practices and predictors of delivery at home after the intervention implemented by Kenya government.

## Methods

### Study area

Kilifi County is one of the 47 counties in Kenya located along the Kenya coastline and covers 12,609.7 km^2^ of land. In 2012 it had a population of 1,217,892, with more than 68 % of the population living below poverty line and the main economic activities being subsistence farming (maize and cassava farming), fishing in the Indian Ocean and tourism [7]. The entire road network covers about 3000 Kms. Only 30 kms of rural roads are tarmacked, the rest are in poor state and mostly impassable especially in rainy seasons [3].

The county has nine level 4 public hospitals, 20 level 3 public health Centres, 197 level 2 public dispensaries, one mission hospital, two private hospitals, one armed forces hospital, five private nursing homes and 107 private clinics. Level 4 public hospitals are the primary hospitals, level 3 are health centres, maternities or nursing homes and level 2 are Dispensaries or clinics [7].

The study was carried out in three health facilities within Kilifi County; Kilifi County hospital (level 4), Ganze health centre and Bamba sub-district hospital (level 3). The three health facilities were picked because of their geographical locations, high volume facilities and evenly cover the study location.

### Study design

This was a facility based cross sectional study interviewing mothers in study health facilities who had brought their children for routine immunization services and delivered within six months prior to commencement of the study. The outcome of interest was childbirth either at home or at a health facility.

### Size of the study

The sample size n was calculated using the formula of Fishers et al. [8]

$$ \begin{array}{ll}\hfill n& = {{\mathrm{z}}^2}_{1\hbox{--} \upalpha}\ \mathrm{P}\ \left(1\hbox{--}\ \mathrm{P}\right)/{\mathrm{d}}^2\hfill \\ {}& = {(1.96)}^2\ (0.56)(0.44)/{(0.05)}^2\hfill \\ {}& = 378.63\hfill \end{array} $$

Where *Z* = standard normal distribution curve value for 95 % CI which is 1.96

*P =* proportion of home deliveries according to KDHS of 2007/08–0.56.

d = absolute precision (0.05)

Attrition of 10 % = 0.1* 379 = 38

Therefore a sample size of 417 mothers (379 + 38) was enough to answer the study question after adjusting for 10 % of attrition.

### Study population

The study population was women of child bearing age from 18–49 years attending the study health facilities during the study period. Women attending the study health facilities who had given birth in the last six months prior to study period and a resident of Kilifi County were screened and those eligible were asked to provide written consent to participate in the study. Mothers with very sick children were excluded in the study. The population of females in reproductive age (15–49 years) in the County was 257,521 (23 %) in 2009 [7]. In 2008/09, 56.2 % of mothers delivered at home without being attended by skilled birth attendant, maternal mortality rate was 488 per 100,000 live births in the County [1].

### Data management and statistical analysis

Trained research assistants were used to collect data from the mothers using structured questionnaires. Every questionnaire was cross-checked for completeness after the interview. After data collection, double entry was done on a password protected Microsoft Access database and exported to STATA 13.1 (College Station, TX, USA) for statistical analysis. The distance from the household to the nearest health facility was categorized into 4 groups; <5, 5 to 10, ≥10 kms and don’t know. Categorical variables were summarized using proportions and associations tested using chi-square or fisher’s exact test where applicable. Continuous variables were summarized using means and standard deviations for normally distributed data while skewed data were summarized using medians and interquartile range. A two tailed independent t-test was used to test difference of means for normally distributed continuous variables and *Mann–Whitney U* test for skewed continuous variables. To identify risk factors of delivering at home, we computed relative risks (RR) using log-binomial regression model, retaining all variables with a crude *P*-value < 0.1 (10 %) in the multivariate model. Statistical significance was evaluated using 95 % confidence interval and a two-tailed *p*-value of <0.05.

### Ethical considerations

The study was approved by Kenya Ethical Review Committee and conducted in accordance to good clinical practices principles. Permission to conduct the study was also granted by Kilifi County Director of Health Services and the study health Facilities in-charges.

## Results

We recruited a total of 410 mothers; 100 from Bamba sub-district hospital, 100 from Ganze health centre and 210 from Kilifi county hospital but only 394 records had data on the outcome of interest and were used in the analysis. Of the 394 mothers, 103 (26 %) reported to have delivered at home during their last pregnancy. The mean age in years of the mothers and their partners (standard deviation) was 25.9 (5.6) and 31.5 (7.3) respectively. Mothers who delivered at home were older (*P =* 0.002). The distribution of marital status and whether the partner was the household main bread winner was not different between mothers who delivered at home and those who delivered at a health facility. The distribution of other main socio-demographic characteristics (type of marriage, number of children, religion and level of education) were different between mothers who delivered at home and those who delivered at a health facility (*P <* 0.05) Table 1.

**Table 1:** Participants characteristics stratified by either home or health facility delivery

Characteristics	Delivered at home (*N =* 103)	Delivered in a health facility (*N =* 291)	Overall (*N =* 394)	*P*-value
Age in years Mean (sd)	27.4 (6.1)	25.4 (5.3)	25.9 (5.6)	0.002
Marital Status *N* (%)				
Married	91 (25)	262 (72)	365 (90)	
Divorced	4 (36)	7 (64)	11 (2.7)	0.43
Widowed	3 (50)	3 (50)	6 (1.5)	
Single/never married	5 (20)	19 (76)	25 (6.1)	
Type of marriage				
Monogamy	75 (23)	244 (74)	328 (80)	
Polygamy	23 (44)	26 (50)	52 (13)	<0.0001
Others	0	13 (100)	13 (3.2)	
Missing	5 (29)	8 (47)	17 (3.2)	
Number of children				
One child	16 (12)	109 (85)	129 (31)	
2 to 4 children	56 (28)	139 (69)	202 (49)	<0.0001
5 children and above	31 (39)	43 (54)	79 (19)	
Religion				
Christian	79 (25)	227 (72)	316 (77)	
Muslim	24 (26)	64 (70)	91 (22)	<0.0001
Level of education				
None	26 (39)	36 (55)	66 (16)	
Primary level	63 (26)	172 (	242 (59)	0.001
Secondary level	12 (18)	54 (82)	66 (16)	
Post-secondary level	2 (6.3)	28 (88)	32 (7.8)	
Partner Characteristics				
Age in years Mean (sd)	33.0 (8.6)	31.0 (6.8)	31.5 (7.3)	0.02
Level of education				
None	13 (43)	16 (53)	30 (7.3)	
Primary level	51 (28)	126 (69)	183 (45)	0.001
Secondary level	29 (24)	91 (74)	123 (30)	
Post-secondary level	6 (13)	39 (85)	46 (11)	
Partner the decision maker	79 (24)	248 (76)	338 (86)	0.02
Partner the bread winner	85 (26)	242 (74)	338 (86)	0.24
Distance from household to the nearest hospital				
<5 kms	26 (15)	151 (85)	179 (44)	
5 to 10 Kms	36 (28)	94 (72)	137 (34)	<0.0001
≥10 Kms	25 (45)	30 (55)	58 (14)	
Don’t Know	16 (52)	15 (48)	32 (8)	

Among the 103 mothers who delivered at home, only 44 (43 %) were massaged on the abdomen during labor. A total of 59 (57 %) of mothers who delivered at home used new washed/unwashed clothes to wrap the baby and 42 (41 %) wrapped the baby with old washed/unwashed clothes. Of the babies delivered at home 46 (44 %) were wrapped within five minutes of delivery and 81 (79 %) were wrapped in the first 30 min. Mother’s breast milk was the common food first fed to the baby 71 (69 %) and 16 (17 %) babies were fed with glucose water first, 81 (79 %) of these babies delivered at home were fed within the first hour of life. Cotton and gauze was used to control bleeding by 35 (36 %) mothers, 47 (48 %) used clean washed/new cloth and 15 (15 %) used unwashed cloth. Eighthly nine (88 %) of mothers who delivered at home went to the hospital after delivery, 59 (65 %) visited the hospital after a day of delivery, 70 (78 %) visited hospital for the baby immunization, 13 (14 %) for check-up and 4 (4 %) because there was complication during home delivery. Among those babies delivered at home and taken to hospital later, 87 (92 %) got vaccinated, 72 (76 %) of the babies were vaccinated after a day. Majority of mothers who deliver at home were assisted by their mother-in-laws (28 %) and only 4.9 % were assisted by a skilled birth attendant Table 2. Despite very few of home deliveries being supervised by skilled birth attendant, 98 (95 %) of mothers who delivered at home used new or boiled blade to cut the umbilical cord.

**Table 2:** Some reasons for delivering at home and home delivery practices among mothers who delivered at home

Characteristics (*N =* 103)	*N* (%)
Why did you deliver at home?	
Onset of labor before the expected date	33 (32)
Hospital is too far	12 (12)
Home delivery is easy & convenient	11 (11)
All my previous deliveries were at home	9 (8.7)
Worries about cost in the hospital	8 (7.8)
Missing	30 (29)
Where exactly at home did you deliver?^a^	
In a room	47 (46)
Inside the house	44 (43)
Outside the house	7 (6.9)
On the way	4 (3.9)
Who assisted or attended to you during delivery?^b^	
Mother-in-law	29 (28)
Mother	19 (18)
Traditional birth attendant	18 (17)
Neighbour	10 (9.7)
No attendant	10 (9.7)
Other family members	6 (5.8)
Skilled birth attendant	5 (4.9)
In what position did you deliver?	
Lying down	43 (42)
Squatting	29 (28)
Kneeling	19 (18)
Others /Standing/Missing	12 (12)
What was the surface of delivery?^b^	
Clean washed cloth/surface	49 (48)
On unwashed cloth/surface	26 (25)
On the ground	22 (21)
Health facility factors made you deliver at home?^c^	
Distance	56 (54)
Health workers attitudes	20 (19)
Cost	12 (12)

^a^-1 missing record, ^b^-6 missing records, ^c^-15 missing records

Living far from a health facility was reported as a reason for delivering at home by 54 % of mothers who delivered at home and 32 % of mothers who delivered at home were not able to get to a health facility because of onset of labor before the expected delivery date. Only 12 % of mothers reported high cost as a reason for not delivering at health facility Table 2.

Although only 68 (17 %) mothers reported to have planned to deliver at home, 103 (26 %) delivered at home. This is despite the fact that 350 (87 %) of all the interviewed mothers acknowledged knowing the difference between delivering at home and in a health facility and that delivering at home was dangerous. Although only 18/103 (17 %) of mothers who delivered at home reported to have been attended by the traditional birth attendant (TBA), 84 (21 %) of all the mothers reported TBA services to be good. A total of 81 (21 %) of all mothers thought TBA services were essential, majority being mothers who delivered at a health facility (*P <* 0.0001). However, 210 (53 %) believed TBA services in managing home deliveries was bad. We found 373 (92 %) of all mothers to have attended antenatal care (ANC) albeit 61 (16 %) started attending ANC in third trimester and only 183 (49 %) attended ANC at least four times as recommended. Majority of mothers who attended the ANC clinics reported their services were good 253 (68 %) and 305 (82 %) reported to have been advised on mode of delivery during the ANC clinic visits Table 3.

**Table 3:** Mothers’ Antenatal Care (ANC) experience and perceptions towards home delivery

Characteristics	Delivered at home (*N =* 103)	Delivered in a health facility (*N =* 291)	Overall (*N =* 394)	*P*-value
In the last pregnancy, did you plan to deliver at home?	35 (34)	30 (10)	65 (17)	<0.0001
In the last pregnancy, did you attend antenatal care?	86 (84)	277 (95)	363 (92)	<0.0001
When did you start antenatal care?^a^				
First trimester	21 (19)	88 (30)	109 (27)	
Second trimester	50 (49)	145 (50)	195 (50)	0.303
Third trimester	16 (16)	43 (15)	59 (15)	
During last pregnancy, how many times did you attend the ANC?^b^				
Once	11 (11)	15 (5.2)	26 (6.6)	
Twice	17 (17)	41 (14)	58 (15)	
Thrice	17 (17)	79 (27)	96 (24)	0.109
4 times	24 (23)	73 (25)	97 (24)	
5 times and above	16 (16)	67 (23)	83 (21)	
During the last pregnancy and in your own opinion, how was the ANC care and treatment?^a^				
Bad	5 (4.9)	5 (1.7)	10 (2.5)	
Good	63 (61	185 (64)	248 (63)	
Better	13 (13)	46 (16)	59 (15)	0.04
Excellent	5 (4.9)	41 (14)	46 (12)	
Advised on the mode of delivery during the ANC visits?	61 (59)	236 (81)	297 (75)	0.007
There is a difference between delivering at home and delivering at the hospital	69 (67)	270 (93)	339 (86)	<0.0001
It is dangerous to deliver at home	68 (66)	270 (93)	338 (86)	<0.0001
What is your view on the services offered by TBA’s in the management of home deliveries?				
Good	45 (44)	37 (13)	82 (21)	
Essential	26 (25)	63 (22)	89 (23)	
Bad	23 (22)	180 (62)	203 (52)	<0.0001
Better	9 (8.7)	11 (3.8)	20 (5.1)	

^a^-missing 32 records, ^b^-missing 34 records

From the univariate analysis, both mother and the partners old age, being in a polygamy marriage, being a mother of at least two children and staying ≥ 5 Kms from the nearest health facility were associated with higher risk of delivering at home (crude *P <* 0.05). However, both higher education level of the mother and the partner were associated with a protective effect on the risk of delivering at home in the univariate regression model (RR < 1.0 and *P <* 0.05). In multivariate regression model, only long distance from the nearest health facility was associated with higher risk of delivering at home. Living ≥10 Kms away from the nearest health facility was associated with adjusted RR of 3.86 (95 % CI 2.13 to 7.02, *P <* 0.0001) Table 4.

**Table 4:** Risk factors associated with home delivery

Variable	Crude Risk ratios (95 % CI)	*P*-value	Adjusted Risk ratios (95 % CI)	*P*-value
Age in years	1.04 (1.02 to 1,07)	<0.0001	1.06 (0.99 to 1.23)	0.10
Partners age in years	1.02 (1.01 to 1.04)	0.009	0.98 (0.93 to 1.03)	0.46
Marital Status				
Married	1.0	Reference		
Divorced	1.41 (0.63 to 3.14)	0.40		
Widowed	1.94 (0.85 to 4.40)	0.11		
Single/never married	0.81 (0.36 to 1.80)	0.60		
Type of marriage				
Monogamy	1.0	Reference	1.0	Reference
Polygamy	1.99 (1.40 to 2.85)	<0.0001	1.30 (0.75 to 2.23)	0.35
Number of children				
One child	1.0	Reference	1.0	Reference
2 to 4 children	2.24 (1.35 to 3.73)	0.002	1.80 (0.92 to 3.50)	0.08
5 children and above	3.27 (1.93 to 5.56)	<0.0001	1.35 (0.56 to 3.26)	0.51
Religion				
Christian	1.0	Reference		
Muslim	1.06 (0.71 to 1.56)	0.78		
Level of education				
None	1.0	Reference	1.0	Reference
Primary level	0.64 (0.45 to 0.92)	0.02	0.89 (0.51 to 1.58)	0.71
Secondary level	0.43 (0.24 to 0.78)	0.005	0.65 (0.27 to 1.59)	0.35
Post-secondary level	0.16 (0.04 to 0.63)	0.009	0.24 (0.05 to 1.25)	0.09
Partners Level of education				
None	1.0	Reference	1.0	Reference
Primary level	0.64 (0.40 to 1.02)	0.06	0.69 (0.34 to 1.42)	0.32
Secondary level	0.54 (0.32 to 0.91)	0.02	0.65 (0.29 to 1.46)	0.29
Post-secondary level	0.30 (0.13 to 0.69)	0.005	0.55 (0.16 to 1.86)	0.34
Distance from household to the nearest hospital				
<5 kms	1.0	Reference	1.0	Reference
5 to 10 Kms	1.89 (1.20 to 2.96)	0.006	1.58 (0.89 to 2.80)	0.12
≥10 Kms	3.09 (1.96 to 4.89)	<0.0001	3.86 (2.13 to 7.02)	<0.0001
Don’t Know	3.51 (2.15 to 5.75)	<0.0001	2.95 (1.48 to 5.90)	0.002

## Discussion

Our study established that 26 % of the mothers attending child welfare clinics for child immunization delivered their last child at home, a prevalence lower than 2014 national prevalence of 39 % and 51 % in Kilifi County [9]. Studies done in other Counties in Kenya like Pokot and Nyandarua reports higher prevalence [10–12]. Our lower proportion of mothers delivering at home could be due to efforts by Kenya government of increasing hospital access to mothers especially in rural areas. Kenya government in collaboration with other health stakeholders has been carrying out high-level campaign and interventions to reduce maternal mortality in line with Millennium Development Goal 5. In particular the current first lady is leading a campaign dubbed “Beyond zero campaign”, to stop preventable maternal deaths by providing fully equipped mobile clinic to provide medical care during delivery to women who have no access to hospitals. By only including mothers bringing their babies for immunization services, we might have left out mothers who don’t take their babies for vaccination or those who lost their infants in the first six months of life.

Previous studies investigating factors associated with place of delivery, reported age of the mother as one of risk factor associated with home delivery [13–16]. In the univariate analysis, both age of the mother and that of the partner were associated with the risk of home delivery. Higher age of the mother would be a function of successful previous deliveries experience and some cultural norms. Mothers who have previously delivered successfully with no complications tend to deliver at home than the young new mothers. On the other hand, older women may belong to more traditional cohorts and thus be less likely to use modern facilities than young women [17]. High education levels of both the partner and the mother were associated with protective effect on the risk of home delivery. Educated women tend to give birth to few children and deliver at a health facility compared to women with little or no education [8, 18, 19].

Contrary to previous hypothesis, cost of delivery at health facility was not reported as a major hindrance to accessing hospital delivery services rather distance from the nearest health facility was the major risk factor of home delivery. After adjusting for other risk factors, staying ≥10 kms from nearest health facility was associated with nearly 4-fold risk of home delivery. This key finding concurs with what other researchers in developing countries have reported [20–26]. Most pregnant women are not able to access transport services when they develop labor mostly due to the poor road net-work and infrastructure especially in rural and poor urban regions in Africa [20, 22]. Within rural Kilifi County, health facilities are sparsely distributed with very poor road net-work and erratic public transport system. Most of the women could have developed labor at night when the public means of transport is not available. Interventions such as “waiting homes” near health facilities to accommodate the expectant mothers residing far from the nearest health facilities days before delivery day can be helpful in such scenarios [19]. Government run health facilities in Kenya, offer free maternal health services but this may not help the targeted mothers if the mothers can’t access the health facilities. The ‘beyond zero initiative’ by the first lady was conceived to reach to these mothers who may not be able to access health facilities to deliver however the initiative is yet to have significant impact on reducing maternal mortality. Despite the fact that Kilifi County is one of the Counties that received the mobile clinics, it has not been able to reach to the rural Kilifi where our study population reside [27].

Majority of the women who delivered at home were assisted by untrained birth attendants like their mother-in-laws or their biological mothers, very few were attended by TBA or skilled birth attendants. This poses great danger to the pregnant woman and the baby since the untrained birth attendants ‘delivery services’ are informed by some detrimental cultural practices like massaging of the abdomen before delivery. Massaging is done to align the baby in the right position in readiness for delivery. Most of the massaged women are unaware of the dangers associated with it that include placenta praevia and abruption, asphyxiating the fetus and increased chances of trauma to the baby and premature delivery. During focus group sessions, mothers complained of many uncomfortable vaginal examinations which could have kept some pregnant women away from the maternity. Mothers delivering at home don’t get the professional advice on how to treat the umbilical cord, it’s a common practice to apply wood ash after cutting the umbilical cord along the Kenya coast. These cultural practices especially by mothers delivering at home are associated with the excess burden of neonatal admissions with such common diagnosis like neonatal sepsis and neonatal Tetanus at Kilifi County Hospital [28, 29].

The major limitation of this study was selection bias since the study was carried out in health facilities excluding mothers who don’t take their children to child welfare clinic and whose children died in the first six months. Mothers of deceased infants and who don’t take their children for immunization might have different reasons for delivering at home. Therefore the proportion of home delivery reported by this study was an underestimation.

## Conclusion

Despite the government efforts to offer free maternal services in Kenya, long distance from the nearest health facility rather than economic and cultural factors was the major reason why mothers still deliver at home. Government should consider investing in innovative ways of increasing access to health facility by pregnant women.

## References

[1] Kenya National Bureau of Statistics (KNBS) and ICF (2010). Macro Kenya Demographic and Health Survey 2008–09.

[2] National Co-ordinating Agency for Population and Development (NCPDA) (2010). Maternal Deaths on the Rise in Kenya: A Call to Save Women’s Lives. Policy Brief No. 9 – June 2010.

[3] Kenya National Bureau of Statistics (KNBS) (2010). Kenya Population and Housing Census Report published in August 2010.

[4] CBS, MOH and ORC Macro (2004). Kenya Demographic and Health Survey,2003.

[5] Kenya Demographic and Health Survey 2014. Available on www.http://dhsprogram.com/pubs/pdf/PR55/PR55.pdf [accessed on 07May2015].

[6] Njuki R, Abuya T, Kimani J, Kanya L, Korongo A, Mukanya C (2015). Does a voucher program improve reproductive health service delivery and access in Kenya?. BMC Health Serv Res.

[7] Kilifi County: Kilifi County Profile Available on: http://www.kilifi.go.ke/index.php?com=1#.VnaoB1In504 [accessed on 20 Dec 2015]

[8] Fisher AA, Laing EJ, Stoeckel EJ, Townsend WJ. Handbook for family planning operations research design. 2nd. New York, NY, USA: Population Council; 1998. p.36.

[9] Kenya National Bureau of Statistics (KNBS) (2014). Kenya Population and Housing Census Report published in April 2015.

[10] Ogolla J. “Factors Associated with Home Delivery in West Pokot County of Kenya,” Advances in Public Health.2015, Article ID 493184, 6 pages,doi:10.1155/493184

[11] Wanjira C, Mwangi M, Mathenge E, Mbugua G, Ng’ang’a Z (2011). Delivery practices and associated factors among mothers seeking child welfare services in selected health facilities in Nyandarua South District Kenya. BMC Public Health.

[12] Mokua J. Factors Influencing Delivery Practices among Pregnant Women in Kenya:A Case of Wareng’ District in UasinGishu County, *International Journal of Innovation and Scientific Research. 2014*, ISSN 2351–8014 Vol. 10 No. 1 Oct. 2014, pp. 50–58

[13] Envuladu EA, Agbo HA, Lassa S, Kigbu JH, Zoakah AI (2013). Factors determining the choice of a place of delivery among pregnant women in Russia village of Jos North, Nigeria: achieving the MDGs 4 and 5. Int J Med Biomed Res.

[14] Van Eijik A, Bles H, Odhiambo F, Ayisi J, Blokland I, Rosen D (2006). Use of antenatal services and delivery care among wome n in rural Western Kenya: a community based survey. J Reproductive Health.

[15] Mrisho M, Schellenberg JA, Mushi K, Obrist B, Mshinda H, Tanner M (2007). Factors affecting home delivery in rural Tanzania. Tropical Med Int Health.

[16] Nanang M, Atabila A (2014). Factors predicting home delivery among women in Bosomtwe-Atwima-Kwanwoma district of Ghana: A case control study. Int J Med Public Health.

[17] Navaneetham K, Dharmalingam A (2006). Utilization of maternal health care services in Southern India. Soc Sci Med.

[18] Mwewa D, Michelo C (2010). Factors associated with home deliveries in a low income rural setting-observations from Nchelenge District Zambia. Med J Zambia.

[19] Abebe F, Berhane Y, Girma B (2012). Factors associated with home delivery in Bahirdar Ethiopia: A case control study. BMC Res Notes.

[20] Shrestha SK, Banu B, Khanom K, Ali L, Thapa N, Stray-Pedersen B (2012). Changing trends on the place of delivery: why do Nepali women give birth at home?. Reprod Health.

[21] Kulmala T. Maternal Health and Pregnancy Outcomes in Rural Malawi. Accademic Dissertation, University of Tempere. Medical School, Acta Electronica Universitatis Temperenasis 76; 2000. Available on http://uta32-kk.lib.helsinki.fi/bitstream/handle/10024/67088/951-44-4976-2.pdf?sequence=1 [accessed on 10 May 2015]

[22] Thaddeus S, Maine D (1994). “Too far to walk: maternal mortality in context,”. Social Science and Medicine.

[23] Doctors of the World USA*.* Partnership for Maternal and Neonatal Health—West Pokot District Child Survival and Health Program. 2007. Available on: http://pdf.usaid.gov/pdf_docs/Pdack178.pdf [accessed on 30 May 2015]

[24] Gabrysch S, Cousens S, Cox J, Campbell O (2011). The Influence of Distance and Level of Care on Delivery Place in Rural Zambia: A Study of Linked National Data in a Geographic Information System. PLoS Med.

[25] Gistane A, Maralign T, Behailu M, Worku A, Wondimagegn T. Prevalence and Associated Factors of Home Delivery in Arbaminch Zuria District, Southern Ethiopia: Community Based Cross Sectional Study. Science J Public Health. 2015;3(1):6–9. doi.10.11648/j.sjph.20150301.12.

[26] Gabrysch S, Campbell OM (2009). Still too far to walk: literature review of the determinants of delivery service use. BMC Pregnancy Childbirth.

[27] BeyondZero. Beyond Zero initiative. Available on: http://www.beyondzero.or.ke/ [accessed on 20 Dec 2015]

[28] Mwaniki MK, Gatakaa HW, Mturi FW, Chesaro CR, Chuma JM, Peshu NM (2010). An increase in the burden of neonatal admissions to a rural district hospital in Kenya over 19 years. BMC Public Health.

[29] Ibinda F, Bauni E, Kariuki SM, Fegan G, Lewa J, Mwikamba M (2015). Incidence and Risk Factors for Neonatal Tetanus in Admissions to Kilifi County Hospital, Kenya. PLoS One.

